# YAP promotes autophagy and progression of gliomas via upregulating HMGB1

**DOI:** 10.1186/s13046-021-01897-8

**Published:** 2021-03-16

**Authors:** Min Zhao, Yu Zhang, Yang Jiang, Kai Wang, Xiang Wang, Ding Zhou, Yan Wang, Rutong Yu, Xiuping Zhou

**Affiliations:** 1 Institute of Nervous System Diseases, Xuzhou Medical University, Xuzhou, 221002 Jiangsu China; 2 Department of Neurosurgery, Affiliated Hospital of Xuzhou Medical University, Xuzhou, 221002 Jiangsu China; 3 The Graduate School, Xuzhou Medical University, Xuzhou, 221002 Jiangsu China; 4 Present address: Clinical Medical College, Yangzhou University, Yangzhou, 225001 Jiangsu China

**Keywords:** Glioma, YAP, Autophagy, HMGB1, Progression

## Abstract

**Background:**

Due to the hypoxia and nutrient deficiency microenvironment, glioblastoma (GBM) exhibits high autophagy activity and autophagy plays an important role in the progression of GBM. However, the molecular mechanism of autophagy in GBM progression remains unclear. The aim of this study is to delve out the role and mechanism of yes-associated protein (YAP) in GBM autophagy and progression.

**Methods:**

The level of autophagy or autophagy flux were assessed by using western blotting, GFP-LC3 puncta (Live) imaging, transmission electron microscopy and GFP-RFP-LC3 assay. The GBM progression was detected by using CCK8, EdU, nude mouse xenograft and Ki67 staining. Isobaric tags for relative and absolute quantification (iTraq) quantitative proteomics was used to find out the mediator of YAP in autophagy. Expression levels of YAP and HMGB1 in tissue samples from GBM patients were examined by Western blotting, tissue microarray and immunohistochemistry.

**Results:**

YAP over-expression enhanced glioma cell autophagy under basal and induced conditions. In addition, blocking autophagy by chloroquine abolished the promoting effect of YAP on glioma growth. Mechanistically, YAP over-expression promoted the transcription and translocation of high mobility group box 1(HMGB1), a well-known regulator of autophagy, from nucleus to cytoplasm. Down-regulation of HMGB1 abolished the promoting effect of YAP on autophagy and glioma growth. Furthermore, the expression of YAP and HMGB1 were positively associated with each other and suggested poor prognosis for clinical GBM.

**Conclusion:**

YAP promoted glioma progression by enhancing HMGB1-mediated autophagy, indicating that YAP-HMGB1 axis was a feasible therapeutic target for GBM. Our study revealed a clinical opportunity involving the combination of chemo-radiotherapy with pharmacological autophagy inhibition for treating GBM patients with YAP high expression.

**Supplementary Information:**

The online version contains supplementary material available at 10.1186/s13046-021-01897-8.

## Background

Glioblastoma (GBM) is the most common and lethal intracranial primary tumor. Despite advances in surgery and adjuvant therapy, the median overall survival time of GBM patients still does not exceed 15 months [1]. Therefore, it is urgent to reveal the molecular mechanism and develop new therapy strategies for GBM.

Autophagy is the process of cellular self-digestion, by which some damaged proteins or organelles are enveloped by double-layered membrane vesicles and targeted to the lysosome for degradation and recycling [2]. Autophagy appears to serve as a pro-survival stress response in most settings by providing energy and metabolic precursors under conditions of starvation and to alleviate stress by removal of damaged proteins and organelles [3–5]. As an important degradation and stress response pathway, autophagy maintains a certain basic level under normal growth conditions, while it increased dramatically under stress challenge [6]. In cancers, autophagy can play neutral, tumor-suppressive, or tumor-promotive roles in different contexts and stages of cancer development [7–9], which is determined by nutrient availability, microenvironment stress and the presence of an immune system. Generally, in established tumor, by enhancing stress tolerance and providing more nutrient and energy, autophagy plays important roles in supporting tumor cell survival [10]. Due to the hypoxia and nutrient deficiency microenvironment, GBM exhibits high autophagy activity and autophagy plays an important role in the progression of GBM [11, 12].

Hippo/YAP pathway is a well-known mechanism that controls organ size by regulating cell growth and proliferation [13]. The mammalian Hippo/YAP pathway is a kinase cascade, which phosphorylates YAP/TAZ, two transcriptional coactivators, ultimately leading to transcription inhibition of the target genes, such as *CTGF* and *CYR61*, and the following cell growth inhibition [14]. Accumulating evidence suggests that Hippo/YAP pathway is dysregulated in many human cancers. Elevated YAP/TAZ expression or nucleus enrichment has been observed in many types of cancers, including liver, breast, lung, colon, ovary and others [15]. We also report that YAP/TAZ is up-regulated in gliomas and YAP promotes glioma progression by inhibiting GSK3β and then activating β-catenin [13, 16, 17]. Recently, growing interest in Hippo/YAP pathway is fueled by studies illustrating that core components of Hippo/YAP signaling are inextricably linked to autophagy. For example, Tang et al. find that LATS1 stabilizes the autophagy core-machinery component Beclin1 by promoting K27-linked ubiquitination at lysine residues K32 and K263 [18]. Hippo kinases MST1/2 sustain autophagosome formation by phosphorylating microtubule-associated protein 1 light chain (LC3B), a marker for autophagy [19]. In addition, YAP enhances autophagic flux in human ovarian and breast cancer cell lines [4, 20] and decreases the sensitivity of cancer cells to chemotherapeutic drugs, such as cisplatin [21]. However, the molecular mechanism of YAP in autophagy has not been fully elucidated.

High mobility group box 1 (HMGB1) is an evolutionarily conserved non-histone DNA-binding protein mainly located in the nucleus, cytoplasm, and extracellular sometimes. According to its level and location, HMGB1 is involved in many physiological and pathological cellular processes, such as DNA damage response, gene transcription, autophagy, cell proliferation, inflammation and immunity [22–24]. Recent studies have reported that HMGB1 promotes autophagy through various signaling pathways in different intracellular and extracellular locations [25]. For example, in nucleus, HMGB1 upregulates the expression of HSP27 to induce autophagy [23]. In cytoplasm, the Beclin1/PI3K-III complex can be activated by HMGB1 to promote autophagy [26, 27]. In this study, we demonstrated that YAP promotes glioma progression by enhancing HMGB1-mediated autophagy, which indicates that YAP-HMGB1 signaling axis may be a molecular therapeutic target for GBM. In addition, our study provides a basis for the development of alternative strategies, such as combination therapy with autophagy inhibitors for YAP high GBM patients.

## Materials and methods

### Cell culture

The human glioma U251 and U87 cell lines were purchased from Shanghai Cell bank, Type Culture Collection Committee, Chinese Academy of Sciences. The cells were cultured in Dulbecco’s modified Eagle’s medium (DMEM) (Invitrogen) with 10% fetal bovine serum (FBS, Invitrogen) and penicillin/streptomycin in a humidified incubator under 95% air and 5% CO2 at 37 °C. For nutrient deprivation (ND), cells were incubated in Earle’s Balanced Salt Solution (EBSS, without glucose) after washing with EBSS twice. For some experiments, cells were treated with Chloroquine (CQ, Sigma, 20 μM) or Rapamycin (Rap, Selleck, 200 nM) for indicated time.

### Plasmids and antibodies

The pCDH empty vector and pCDH-YAP^WT^ constructs were generously gifted by Professor Hongbin Ji (Shanghai Institutes for Biological Sciences) [28]. The pEGFP-LC3 is a gift from Zhaotao Wang (General Hospital of Beijing Military Region). The pEGFP-RFP-LC3 plasmid is kindly gifted by Songshu Meng (Dalian Medical University Cancer Center) [20]. EGFP- shHMGB1, mCherry-shHMGB1 and control vector were constructed by Shanghai Genechem Company. The pCDH-CMV-EF1-puro-copGFP-puro-Luciferase (GFP-Luci) is stored in our laboratory [29].

Rabbit monoclonal anti-YAP, rabbit monoclonal anti-HMGB1, rabbit monoclonal anti-Histone and mouse monoclonal anti-p62 were bought from Abcam. Mouse monoclonal anti-β-actin was bought from Millipore. Rabbit monoclonal anti-LC3 was bought from CST. Mouse monoclonal anti-GAPDH was bought from Santa Cruz.

### Transient transfection and lentiviral transduction

GFP-LC3 or GFP-RFP-LC3 was transiently transfected into cells using Lipofectamine 2000 (Invitrogen) according to the manufacturer’s instructions. All the transfections were performed 3 times independently. YAP over-expression or HMGB1 down-regulation glioma cells was generated by lentiviral transduction. Briefly, the pCDH-CMV-EF1-YAP1 construct or control vector, together with packaging plasmids, was transfected into HEK-293 T cells. Forty-eight hours after transfection, the progeny viruses released from HEK-293 T cells were collected, filtered, and were used to infect U251 and U87 glioma cells. Short hairpin (sh)-HMGB1 were ligated in the lentiviral vector of hU6-MCS-Ubiquitin-EGFP-IRES or hU6-MCS-Ubiquitin-Cherry-IRES with a puromycin resistant region. The transduction process was similar to that of YAP.

### Cellular fractionation and western blotting (WB)

Cellular fractionation was conducted by using membrane and cytosol protein extraction kit (Biovision), according to the instruction of manufacturer [13]. Equal amount of protein lysates were subjected to 12% sodium dodecyl sulfate-polyacrylamide gel electrophoresis, transferred to 0.45 μm pore size PVDF membrane (Millipore), and probed with primary antibodies at 4 °C overnight and secondary antibodies at room temperature for 1 h. Bound antibodies were detected by the ECL plus western blotting substrate (Thermo Fisher Scientific, Inc.) and exposed to X-ray films or by ChemiDoc Touch (BIO-RAD). Band densities were quantified by ImageJ software (Wayne Rasband, National Institutes of Health). The relative amount of proteins was determined by normalizing the densitometry value of interest to that of the loading control.

### Transmission electron microscopy (TEM)

The cells were fixed using 2.5% glutaraldehyde in 0.1 M phosphate buffer for 4 h at 4 °C, and then post fixed in 1% osmium tetroxide for 3 h. The samples were scraped and pelleted, dehydrated in a graded series of ethanol baths, infiltrated, and embedded in epon resin. Ultrathin sections (70 nm) were obtained and stained with uranyl acetate for 3 min, and examined using a FEI transmission electron microscope.

### Live-imaging

Cells were cultured in chamber on the platform of EVOS FL Auto cell imaging system.

Images of each group with four randomly selected fields were collected as one frame per 15 min and the recording last for 6 h to make movies. The video was played at 6 frames per second.

### iTraq labeling and LC-MS/MS analysis

The process of isobaric tags for relative and absolute quantification (iTraq) quantitative proteomics was as follows: protein extraction, enzymolysis, iTraq labeling, sample mixing, high performance liquid chromatography (HPLC) separation and liquid chromatography coupled with tandem mass spectrometry (LC-MS/MS) analysis. All the proteins with a false discovery rate (FDR) less than 1% will proceed with downstream analysis.

### Real-time PCR

After RNA isolation, cDNA was synthesized using a First-strand cDNA Kit (Roche) following the manufacturer’s instructions [30]. The cDNA products were amplified using the Fast Start Universal SYBR Green Master Mix (Roche). Amplifications were carried out using the Applied Bio-systems 7500. To quantify gene expression changes, the 2^-△△Ct^ method was used to calculate relative fold-changes after normalizing to the value of β-actin [16]. The PCR primers of HMGB1 were as follows:

- Forward:5′-GCTCCATAGAGACAGCGCCGGG-3′;
- Reverse:5′-CCTCAGCGAGGCACAGAGTCGC-3′

### EdU incorporation assay and CCK8 assay

The cell growth was estimated by EdU incorporation assay (RiboBio) and Cell Counting Kit-8 (CCK8) assay (Beyotime) according to the manufacturer’s instruction and our previous study [30].

### Intracranial glioma model and in vivo imaging analysis in nude mice

All animal experiments were performed according to the guidelines for the care and use of laboratory animals and were approved by the Institutional Animal Care and Use Committee of Xuzhou Medical University. Female athymic BALB/c nude mice aged 4 weeks and weighing 20 g were purchased from Charles River (Beijing, China). The intracranial model of glioma in nude mice was performed according to our previous study [30]. Briefly, YAP over-expression or control U87-GFP-luci cells (1 × 10^6^) in 5 μL Leibovitz’s L-15 medium (Gibco, USA) were injected into the right striatum of athymic nude mice (*n* ≥ 7 per group). At the designated day after transplantation, the intensity of luciferase fluorescence of the nude mice was observed under the Xenogen IVIS Spectrum optical imaging device (Caliper, USA).

For some experiments, CQ (25 mg/Kg) were treated by intraperitoneal injection every other day from day 3 after implantation. To obtain the survival curve, the mice were sacrificed when they appeared hemiplegia, listlessness, cachexia and other neurological symptoms. The cryosections of brain were subjected to hematoxylin and eosin (HE) staining and the tumor volume was calculated according to the formula V = 0.5 × ab^2^ with ‘a’ representing the longest diameter and ‘b’ representing the shortest diameter.

### Immunofluorescence

U87 cells were seeded on coverslip at a 70% cell density for 12 h. The cells were washed with PBS and fixed in 4% PFA for 30 min followed by permeabilization with 0.5% Triton X-100 for 30 min. Thereafter, cells were incubated in 5% BSA for 2 h and probed with primary antibodies at 4 °C overnight. The nuclei were labeled with DAPI and cells were embedded in glycerin and photographed by confocal microscopy (Zeiss 710).

Intracranial glioma slices were incubated with the anti-Ki67 antibody overnight at 4 °C and then incubated with the secondary antibody at room temperature for 1 h. The nuclei were labeled with DAPI, and slices were embedded in glycerin and photographed with confocal microscopy (Zeiss 710).

### Clinical datasets and tissue specimens

In the present study, all datasets were from the following public websites: The Cancer Genome Atlas (TCGA) (https://cancergenome.nih.gov/) and The Chinese Glioma Genome Atlas (CGGA) (http://www.cgga.org.cn/).

We totally collected three cohorts of glioma samples. The first one included 36 cases (nontumor = 9, glioma = 27) to detect the expression level of YAP and HMGB1 by using WB (Fig. 4d-f). Cohort 2 included 20 fresh samples for WB (Fig. 7a-c) and cohort 3 was 51 paraffin-embedded tissues for immunohistochemistry (Fig. 7d-h). The clinical and pathological features of cohort 2 and 3 were showed in Table 1. Among them, only 39 specimens had follow-up information for survival analysis. All of the glioma samples and nontumor brain tissues (decompressive surgery) were obtained from Affiliated Hospital of Xuzhou Medical University. All of the glioma samples were histologically confirmed by a pathologist according to the World Health Organization grading system. Informed consent was obtained from patients involved in this study, and the study protocol was approved by the Clinical Research Ethics Committee of the Affiliated Hospital of Xuzhou Medical University.

**Table 1:** Some information of samples used in Fig. 7 of this study

Clinical/pathological features	Items	Number(%)
Cases	–	71 (100%)
Age	< 60	38 (54%)
	> = 60	33 (46%)
Sex	male	40 (56%)
	female	31 (44%)
Epilepsy	with	55 (77%)
	without	16 (23%)
Intracranial hypertension	with	42 (59%)
	without	29 (41%)
Disorders of consciousness	with	52 (73%)
	without	19 (27%)
Grade	III	28 (39%)
	IV	43 (61%)
Western blotting	–	20 (28%)
IHC	–	51 (72%)
Follow-up information	–	39 (55%)

### Tissue microarray and immunohistochemistry

The tissue microarray was constructed as described previously [30]. Briefly, after having been verified with HE staining, two core punch samples with 1.0 mm diameter were taken from the center of tumor foci to build tissue microarray slides.

Immunohistochemistry was performed according to our previous study [30] . Briefly, slides were incubated at 4 °C overnight with anti-YAP or anti-HMGB1 antibodies (1:200 dilution). Thereafter, sections were developed using a peroxidase substrate 3,3′-diaminobenzidine kit (Beijing Zhongshan Golden Bridge Bio) and counter stained with hematoxylin to stain the nucleus.

### Statistical analysis

The results were representative of experiments repeated at least three times and presented as mean ± SD. Statistical comparisons of data were performed using Student’s *t*-test with 2 tails or one-way ANOVA for multiple comparisons followed by Dunnett’s *t*-test for post hoc pairwise comparisons. Overall survival curves were calculated using the Kaplan-Meier method and compared using the log-rank test. Statistical significance was determined with *p* < 0.05.

## Results

### YAP enhances glioma cell autophagy under basal conditions

During the development of autophagy, the most widely studied autophagy-related protein LC3B was subsequently processed from LC3-I into LC3-II, which was modified with phosphatidylethanolamine and bound to the surface of the autophagic vacuole membrane. Therefore, located at the pre-autophagic and autophagic bodies, LC3-II is widely regarded as a marker of autophagy induction and its sustained accumulation is a reflective of autophagy enhancement. In addition, the selective autophagy cargo p62 is degraded during the proper execution of autophagy, and its accumulation can be used as a marker for inhibition of autophagy [31].

**Fig. 1:**
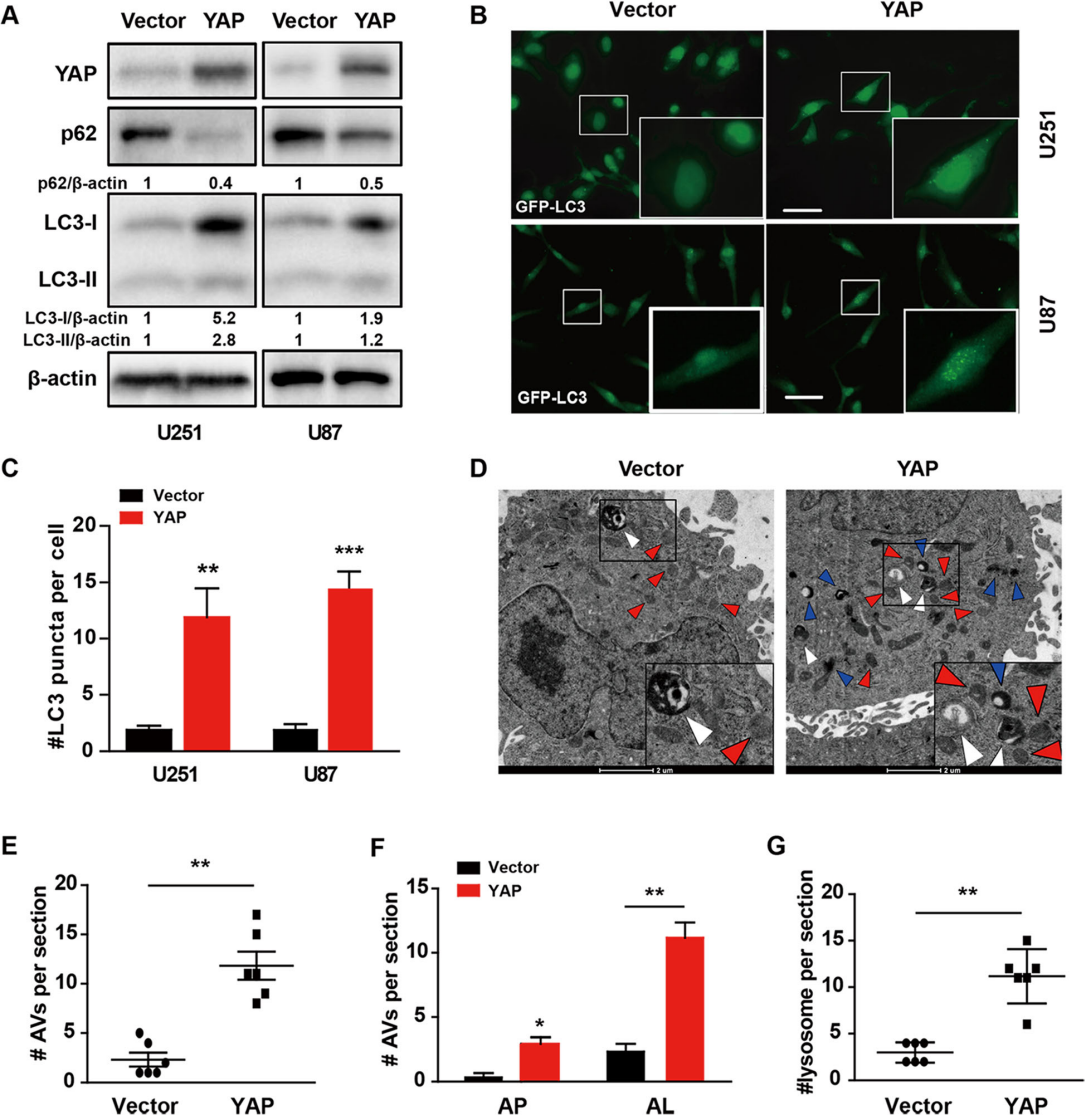
YAP enhances autophagy under basal conditions. **a** Representative immunoblots of LC3 and p62 levels in vector and YAP over-expression U251 and U87 cells under basal conditions. **b** Representative fluorescence micrographs of vector and YAP over-expression U251 and U87 cells expressing GFP-LC3. Scale bar: 50 μm. **c** Quantification of GFP-LC3 puncta presented in (**b**). **d** Representative electron micrographs of vector and YAP over-expression U251 cells. White arrowhead indicated autophagic structure with double-membrane, known as autophagosome (AP); Blue arrowhead indicated autophagic vesicles (AVs) with single-membrane containing cytosolic material undergoing degradation, known as autolysosome (AL); Red arrowhead indicated lysosome. Scale bar: 2 μm. Quantification of AVs (**e**, including AP and AL), AP and AL(**f**) and lysosomes(**g**) in vector and YAP over-expression U251 cells presented in (**d**). The results were representative of experiments repeated at least three times and presented as mean ± SD. *p < 0.05, **p < 0.01, ***p < 0.001

To investigate the role of YAP in autophagy, we firstly examined whether YAP could affect the protein level of LC3-II and p62 in glioma cells. As shown in Fig. 1a, compared with vector control cells, both the protein level of LC3-II and its precursor LC3-I increased, while the p62 level decreased after YAP over-expression. Next, the number of GFP-LC3 puncta increased clearly after YAP over-expression (Fig. 1b & c), indicating that YAP promoted the autophagosome formation both in U251 and U87 cells. In addition, examined by transmission electron microscopy (TEM), the golden standard for detecting autophagy, we found an increase of autophagic vesicles (AVs), autophagosomes (APs) and autolysosomes (ALs) in YAP over-expression U251 glioma cells (Fig. 1d-f). Interestingly, the number of lysosome also increased obviously after YAP over-expression (Fig. 1g), indicating that YAP promotes the maturation and degradation of autophagosomes. Together, the above results showed that YAP enhances glioma cell autophagy under basal conditions.

### YAP enhances autophagy under rapamycin- or starvation-induced conditions

**Fig. 2:**
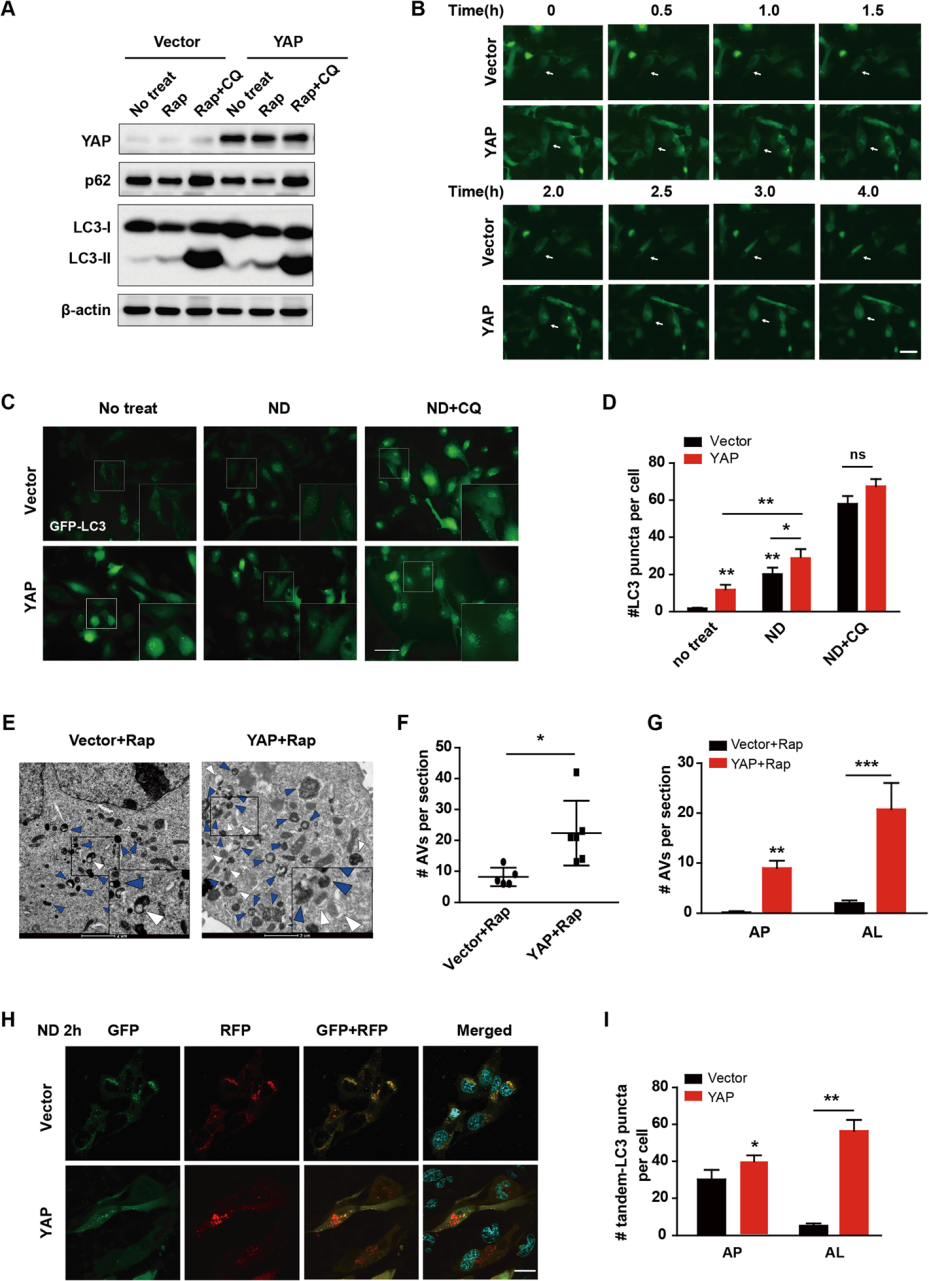
YAP enhances autophagy under rapamycin- or starvation- induced conditions. **a** Representative immunoblots of LC3 and p62 levels in vector and YAP over-expression U251 cells with or without Rap or Rap plus CQ treatment. **b** After having been transfected with GFP-LC3 for 24 h, fluorescence micrographs of vector and YAP over-expression cells were taken at indicated time and representative images were shown. Scale bar: 50 μm. **c** Cells were cultured in normal medium (no treat), EBSS (ND) or EBSS+CQ (ND + CQ) for 2 h. Representative fluorescence micrographs of vector and YAP cells expressing GFP-LC3. Scale bar: 50 μm. **d** Quantification of GFP-LC3 puncta in U251 cells presented in (**c**). **e** Representative electron micrographs of vector and YAP over-expression U251 cells treated with Rap. White arrowhead indicated AP; Blue arrowhead indicated AL. Scale bar: 2 μm. **f** & **g** Quantification of total AVs **(f)**, AP and AL **(g)** of U251 cells presented in (**e**). **h** Having been transfected with pEGFP-RFP-LC3 for 24 h, cells were cultured for 2 h in EBSS (ND 2 h). Representative confocal fluorescence micrographs of vector and YAP over-expression U251 cells. Scale bar: 20 μm. **i** Quantification result of U251 cells presented in (**h**). The results were representative of experiments repeated at least three times and presented as mean ± SD.*p < 0.05, **p < 0.01, ***p < 0.001

To determine whether YAP affects the induced autophagy, we treated U251 cells with Rap (200 nM) with or without autophagy inhibitor CQ (20 μM) treatment. As shown in Fig. 2a, in the vector control cells, the protein level of LC3-II increased, while that of p62 decreased after rapamycin induction. More importantly, YAP over-expression amplified the above results significantly and CQ treatment blocked it. Since autophagy is a dynamic process, we used live-cell imaging to monitor the autophagy process of GFP-LC3 transfecting cells continually after rapamycin induction. As seen in supporting movie 1, 2 and Fig. 2b, YAP over-expression cells displayed more GFP-LC3 clusters than those of the vector cells. In addition, the speed of GFP-LC3 puncta formation and disappearance in YAP over-expression cells was faster than that in vector cells. Consistently, the GFP-LC3 puncta of YAP over-expression cells and vector cells increased dramatically after nutrient deprivation (ND) induction, and YAP over-expression cells had more GFP-LC3 puncta than that of vector cells. Furthermore, CQ treatment eliminated the autophagic flux difference between vector and YAP over-expression cells (Fig. 2c & d). In agreement with our findings by live imaging, the TEM results showed that YAP over-expression cells contained more autophagic vesicles (Fig. 2e & f). Both the APs and ALs increased after rapamycin treatment in YAP over-expression cells and the ALs increased higher than that of APs (Fig. 2g), indicating that YAP promotes the maturation of induced autophagosomes.

To further elucidate that YAP regulates the autophagosome maturation, the pEGFP-RFP-LC3 plasmid was transiently transfected into YAP over-expression U251 cells and the images of APs and ALs were taken with a confocal microscope after ND induction. The GFP signal is pH sensitive and will quenched in the acidic autolysosome, whereas RFP is more stable. Therefore, co-localization of both GFP and RFP fluorescence indicates an AP that has not fused with a lysosome. In contrast, an RFP signal without GFP corresponds to an AL. After being treated with ND for 2 h, YAP over-expression U251 cells displayed less numbers of APs than those of the vector cells slightly, whereas ALs increased significantly (Fig. 2h, i), indicating that YAP promotes the autophagosome maturation by speeding up the process in which AP is fused with lysosome to form AL. Together, YAP enhances autophagy under rapamycin- or starvation-induced conditions.

### Autophagy is crucial for YAP to promote glioma progression

**Fig. 3:**
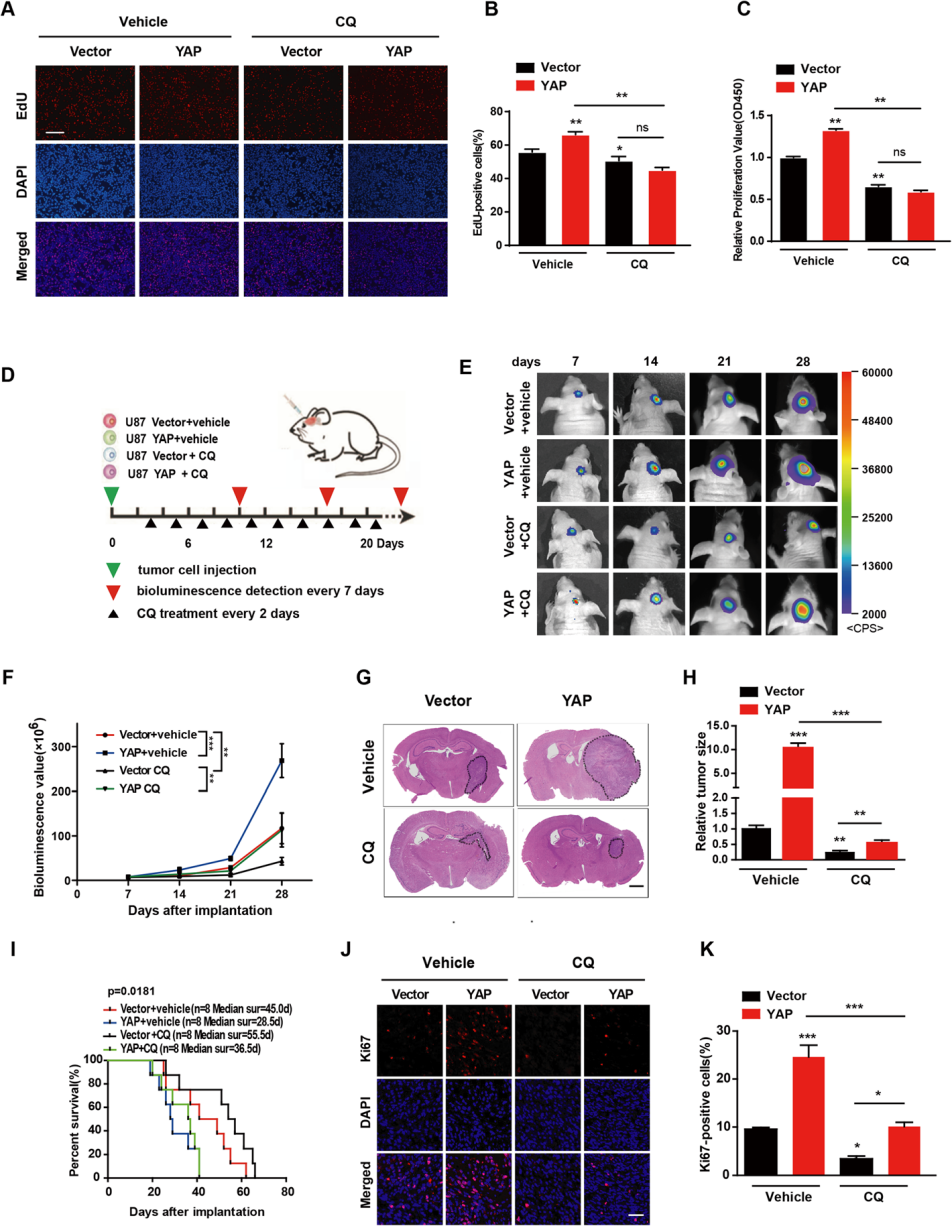
Autophagy is crucial for YAP to promote glioma progression. **a** Representative EdU analysis of cell proliferation in vector and YAP over-expression U251 cells with or without CQ treatment for 14 h. DAPI (blue) was used to stain nucleus and EdU (red) showed the incorporated cells. Scale bar: 200 μm. **b** Quantitative analysis of the EdU positive cells. **c** The effect of CQ treatment on cell proliferation was detected by CCK-8 assay in the vector and YAP over-expression cells. **d** Schematic representation of the in vivo experimental workflow. **e** Representative pseudocolor bioluminescence images of each group at indicated day after implantation. **f** Quantitative analysis of the photon flux. **g** & **h** Representative images of HE staining of tumors derived from vector and YAP over-expression U87 cells with or without CQ treatment (**g**) and quantitative analysis of the tumor volume (**h**). **i** Overall survival was determined by Kaplan-Meier survival curves and a log-rank test was used to assess the statistical significance of the differences (*n* = 8). **j** & **k** Representative images of Ki67 staining of tumors derived from vector and YAP over-expression cells with or without CQ treatment (**j**) and quantitative analysis of the Ki67-positive cells (**k**). Scale bar: 50 μm. All data are expressed as the mean ± SD of values from experiments performed in triplicate. *p < 0.05, **p < 0.01, ***p < 0.001

Since YAP promotes glioma progression [13] and cell autophagy, we wondered whether the promoting effect of YAP on glioma progression is mediated by autophagy. As shown in Fig. 3a & b, examined by EdU incorporation assay, autophagy inhibitor CQ not only significantly inhibited the proliferation of U251 glioma cells, but also abolished the promotion effect of YAP on glioma cell proliferation. Similar result was obtained by CCK8 assay (Fig. 3c).

In addition, we performed the above experiments in intracranial glioma model after injection of the vector control or YAP over-expression U87-GFP-luci cells into the right striatum of athymic nude mice combined with or without CQ (25 mg/kg, every other day) treatment (Fig. 3d). Tumor growth was monitored every 7 days using bioluminescence values. The bioluminescence imaging analyses showed that tumors derived from YAP over-expression cells were significantly bigger than those of vector mice. Importantly, CQ can inhibit the growth advantage of YAP over-expression (Fig. 3e-3h). In addition, Kaplan-Meier survival curve analysis showed that the median survival time of mice injected with YAP over-expression cells were prolonged after CQ treatment, indicating that CQ-treated mice had a survival advantage (Fig. 3i). Consistently, the number of Ki67, the marker for proliferation, positive cells of CQ treatment groups decreased significantly (Fig. 3j & k).

### YAP upregulates HMGB1 and promotes its translocation from nucleus to cytoplasm

**Fig. 4:**
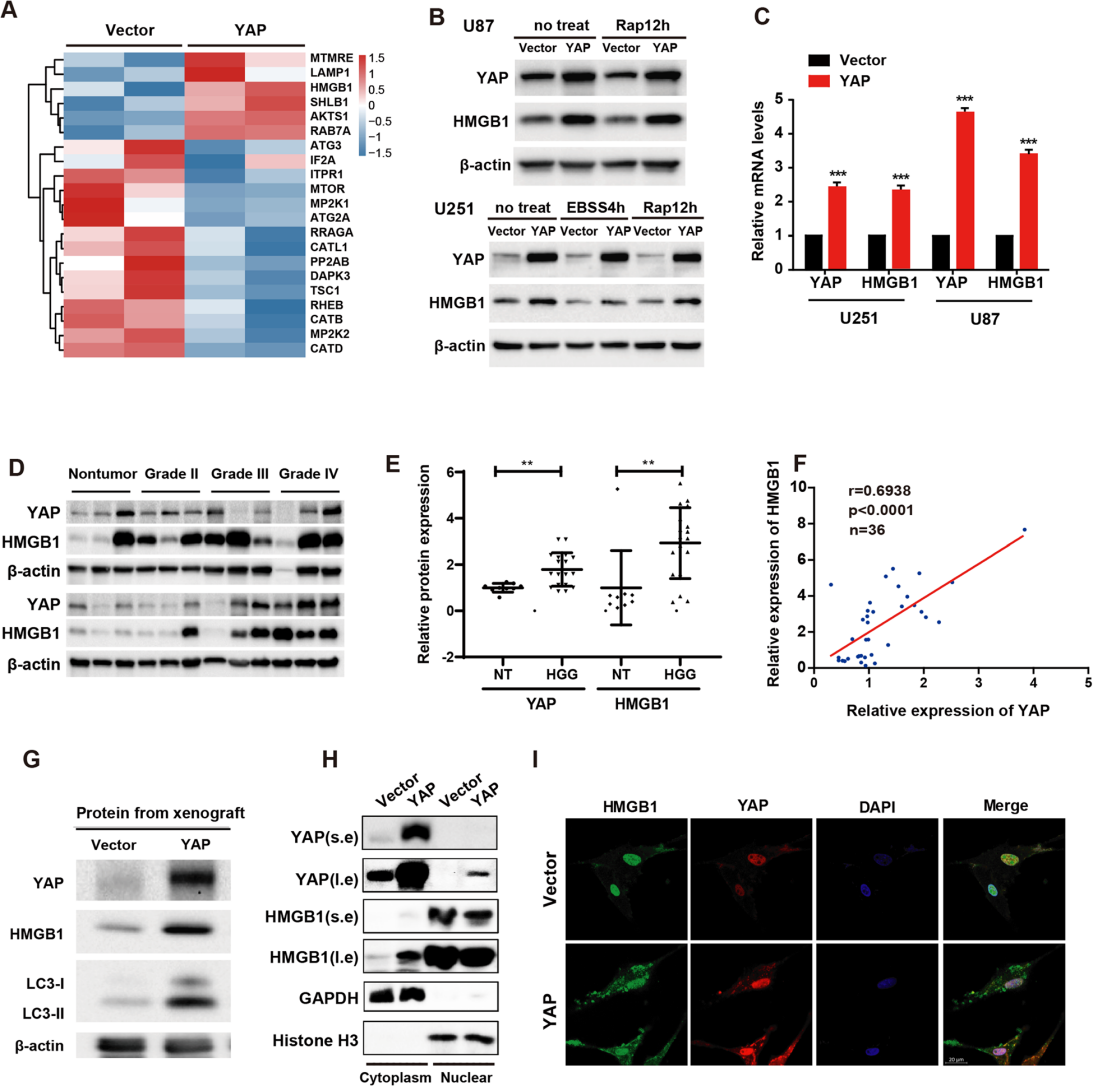
YAP upregulates HMGB1 and promotes its translocation from nucleus to cytoplasm. **a** Heatmap showed the upregulated or downregulated proteins, which is associated with autophagy, after YAP over-expression in U87 glioma cells. **b** Representative immunoblots of the U87 and U251 cell extracts of the vector and YAP over-expression cells with indicated treatment probed with anti-YAP and anti-HMGB1 antibodies. **c** The mRNA level of HMGB1 after YAP over-expression both in U251 and U87 glioma cells. **d** Representative immunoblots of total lysates extracted from nontumor (*n* = 9) or different grade glioma tissues (*n* = 27) probed with indicated antibodies. **e** Quantitative analysis of the protein level of YAP and HMGB1 in gliomas. **f** The protein level of YAP and HMGB1 showed positive correlation in gliomas. *r* = 0.6938, *p* < 0.0001, *n* = 36. **g** Representative immunoblots of the extracts from tumors derived from vector and YAP over-expression cells without CQ treatment probed with indicated antibodies. **h** Subcellular location of HMGB1 was detected by using cellular fractionation. Histone and GAPDH were used as nucleus and cytoplasm loading control respectively. s.e: short exposure. l.e: long exposure. **i** Subcellular location of HMGB1 was assessed by immunofluorescence. Scale bar: 20 μm. *p < 0.05, **p < 0.01, ***p < 0.001

To explore the molecular mechanism of YAP promoting autophagy, we next used iTraq based proteomic analysis to screen for proteins which possibly mediates the promoting effect of YAP on glioma autophagy. According to the proteomic strategy, 21 proteins were defined from YAP over-expression and control comparisons, which satisfied the following two criteria simultaneously: 1) *p* < 0.05 (Mann Whitney Test); 2) belonging to KEGG-Animal-autophagy pathway. Heatmap displayed the relative level of the 21 differentially expressed proteins between two groups (Fig. 4a). Among these proteins, HMGB1 was the most attracting one according to the literature [26, 27]. Therefore, we firstly examined the protein and mRNA levels of HMGB1 after YAP over-expression and found that both the protein and mRNA levels of HMGB1 increased after YAP over-expression (Fig. 4b-c). In addition, because the ubiquitin-proteasome system and autophagy are two major intracellular degradation pathways in eukaryotic cells, we further wonder whether YAP up-regulate HMGB1 protein level by regulating these two pathways. We found that the HMGB1 protein level did not change after YAP over-expression either with MG132 (the proteosome inhibitor, sFig. 1A,1B) or with chloroquine (the autophagy inhibitor, sFig. 1C,1D) treatment, indicating that YAP upregulates HMGB1 not through posttranslational way. Consistent with the above results, the expression of YAP and HMGB1 were upregulated in glioma tissues (Fig. 4d & e, sFig. 2) and positively correlated with each other (Fig. 4f, *p* < 0.001, *r* = 0.6938). Interestingly, the protein level of HMGB1 and LC3-II increased in the xenograft of YAP over-expression mice (Fig. 4g). Importantly, HMGB1 was mainly present in the nucleus, while its level in the cytoplasm increased after YAP over-expression (Fig. 4h & i). Together, the above results indicated that YAP upregulates HMGB1 and promotes its translocation from nucleus to cytoplasm, where it increased the autophagy.

### HMGB1 mediates the promoting effect of YAP on glioma autophagy and growth

**Fig. 5:**
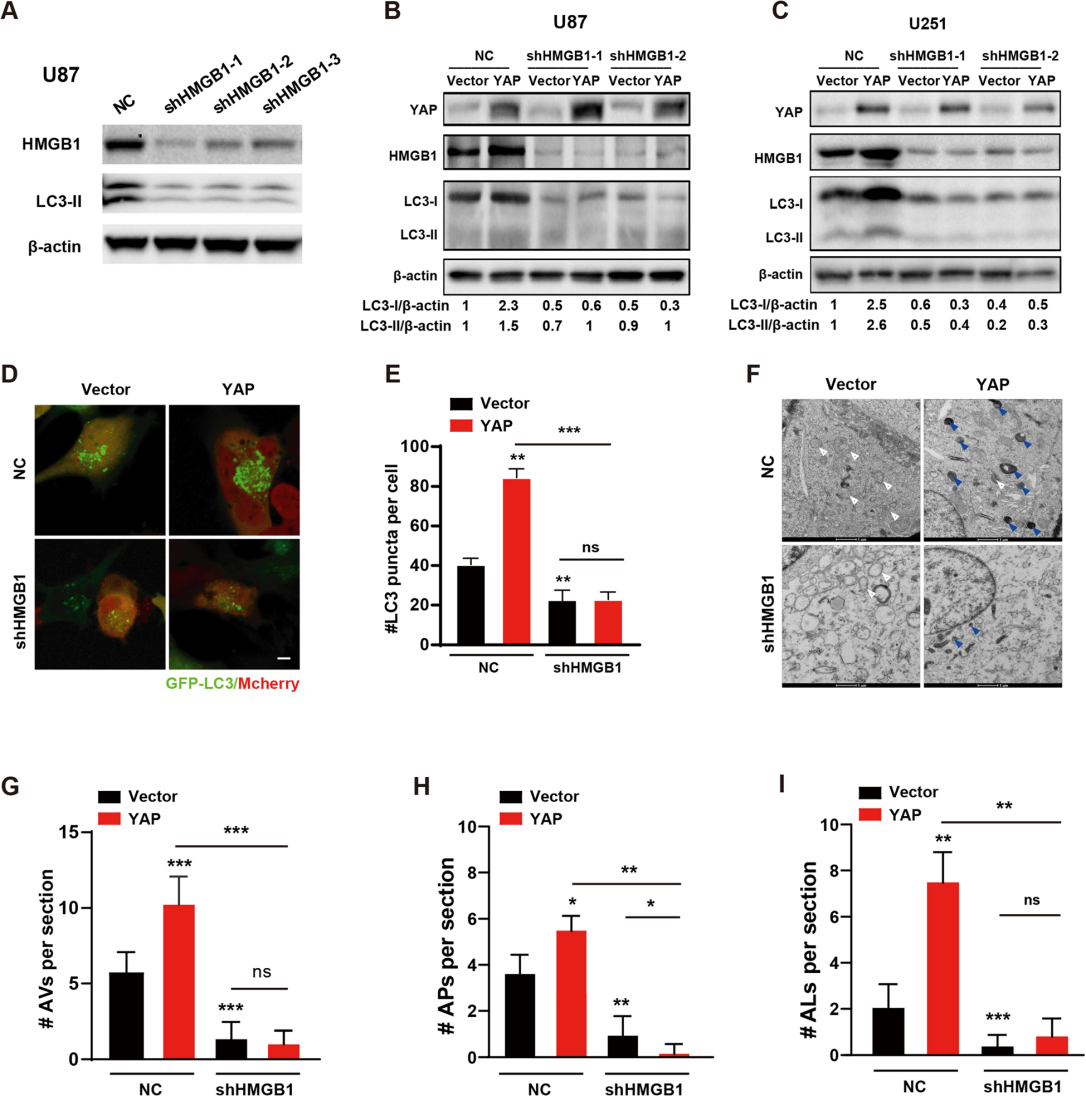
The promoting effect of YAP on autophagy is mediated by HMGB1. **a** Representative immunoblots of the extracts from U87 cells after HMGB1 down-regulation probed with indicated antibodies. **b** & **c** Representative immunoblots of the extracts from U87 (**b**) or U251 (**c**) cells after YAP over-expression with or without HMGB1 down-regulation probed with indicated antibodies. **d** & **e** After having been transfected with GFP-LC3 in U251 glioma cells, representative fluorescence micrographs (**d**) of vector and YAP over-expression cells with or without mCherry-shHMGB1 infection were taken and quantified (**e**). Scale bar, 5 μm. **f** Representative electron micrographs of vector and YAP over-expression U251 cells with or without HMGB1 down-regulation. White arrowhead indicated AP; Blue arrowhead indicated AL. Scale bar: 2 μm. **g** & **h** Quantification of total AVs (**g**), AP (**h**) and AL (**i**) of U251 cells presented in (**f**). All data are expressed as the mean ± SD of values from experiments performed in triplicate. *p < 0.05, **p < 0.01, ***p < 0.001

To examine whether HMGB1 was the mediator of YAP on autophagy, we down-regulated HMGB1 by using three shHMGB1 (sFig. 3A) and found that the protein level of LC3-II decreased after HMGB1 down-regulation (Fig. 5a). Consistently, the effect of YAP on LC3-II was abolished by HMGB1 down-regulation (Fig. 5b & c). In addition, knocking-down of HMGB1 inhibited the increase of GFP-LC3 puncta (mCherry indicated the HMGB1 down-regulating cells, Fig. 5d & e), AVs, APs and ALs induced by YAP over-expression (Fig. 5f-i), indicating that HMGB1 mediates the promoting effect of YAP on autophagy.

**Fig. 6:**
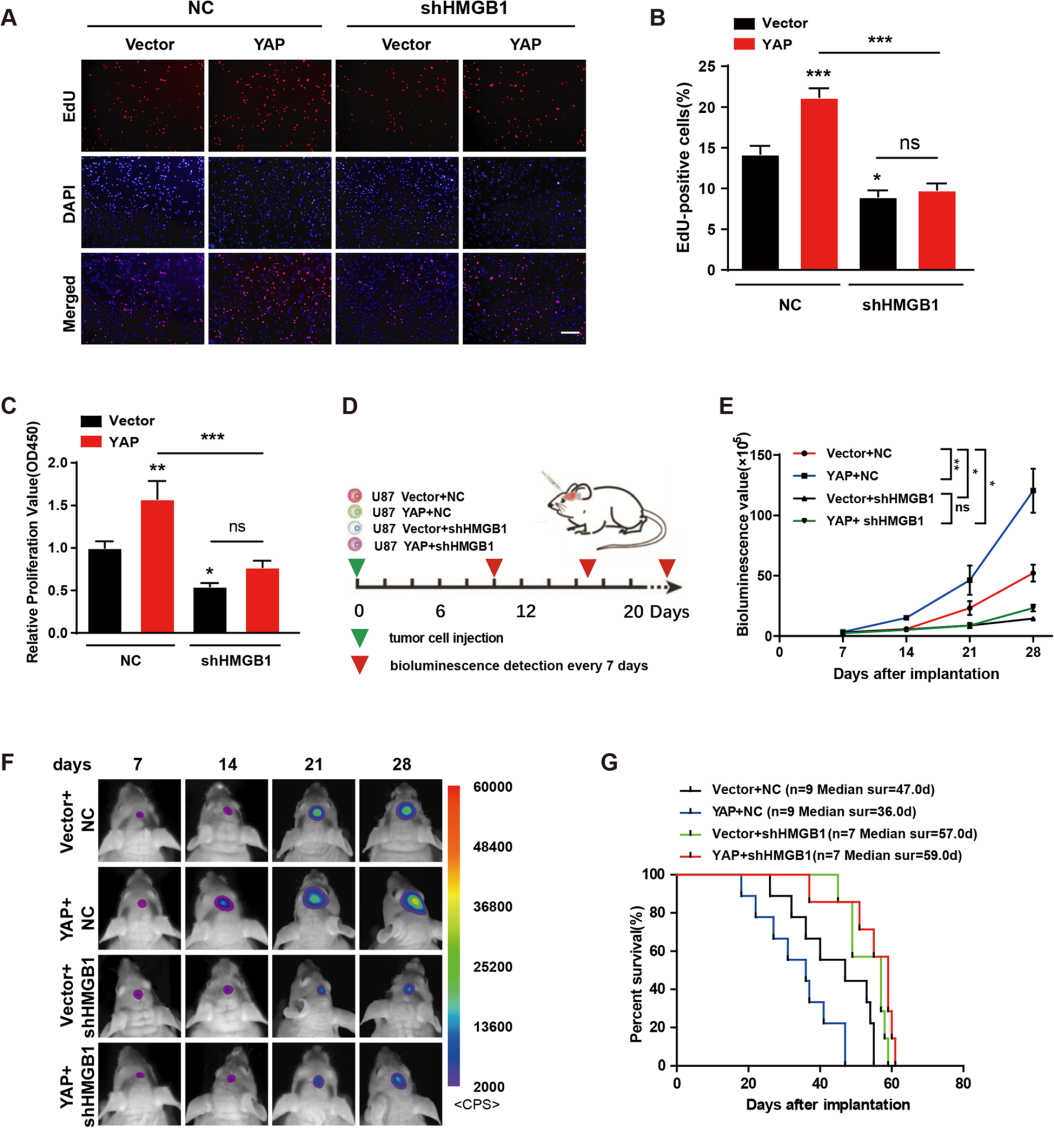
HMGB1 mediates the promoting effect of YAP on glioma growth. **a** Representative images of EdU assay of the vector and YAP over-expression cells with or without HMGB1 down-regulation. Scale bar: 100 μm. **b** Quantification results of (**a**). **c** The effect of HMGB1 knockdown on cell proliferation was detected by CCK-8 assay in the vector and YAP over-expression cells. **d** Schematic illustration of the in vivo experimental workflow. **e** Representative pseudocolor bioluminescence images of each group at indicated day after implantation. **f** Quantitative analysis of the photon flux. **g** Overall survival was determined by Kaplan-Meier survival curves and a log-rank test was used to assess the statistical significance of the differences (*n* ≥ 7). All data are expressed as the mean ± SD of values from experiments performed in triplicate. *p < 0.05, **p < 0.01, ***p < 0.001

We next investigated whether the promotion effect of YAP on glioma growth was mediated by HMGB1. As shown in Fig. 6a & b, examined by EdU incorporation assay, down-regulation of HMGB1 significantly inhibited the viability of U251 glioma cells, and also abolished the promotion effect of YAP. Similar result was obtained by using CCK8 assay (Fig. 6c). Next, the YAP over-expression U87 GFP-Luci cells with or without HMGB1 down-regulation were injected into the right striatum of athymic nude mice to establish intracranial glioma and tumor growth was monitored every 7 days using bioluminescence values (Fig. 6d). The bioluminescence imaging analyses showed that HMGB1 down-regulation markedly inhibited YAP promotion effect on tumor growth (Fig. 6e & f). Similarly, compared with the YAP over-expression group, the median survival time was also prolonged in mice with shHMGB1 and YAP co-expressing xenografts (Fig. 6g). The above results indicate that the promotion effect of YAP on glioma growth was mediated by HMGB1, in line with the result of in vitro experiments.

### Correlative expression of YAP and HMGB1 are prognosis for clinical GBM

To gain insight into the clinical significance of YAP and HMGB1 in glioma samples, we firstly analyzed TCGA and CGGA database. The cut-off to define high or low expression was the median value. During the follow-up period, the overall survival time of patients with low YAP expression was obviously longer than those with high YAP expression (sFig. 4A, CGGA: YAP high, *n* = 334; YAP low, *n* = 334; *p* < 0.0001; sFig. 4B, TCGA: YAP high, *n* = 351; YAP low, *n* = 351; *p* < 0.0001). Moreover, the mRNA level of HMGB1 was also tightly associated with bad prognosis in database of High Grade Glioma (sFig. 4C, CGGA: HMGB1 high, *n* = 244; HMGB1 low, *n* = 244; *p* = 0.0441).

**Fig. 7:**
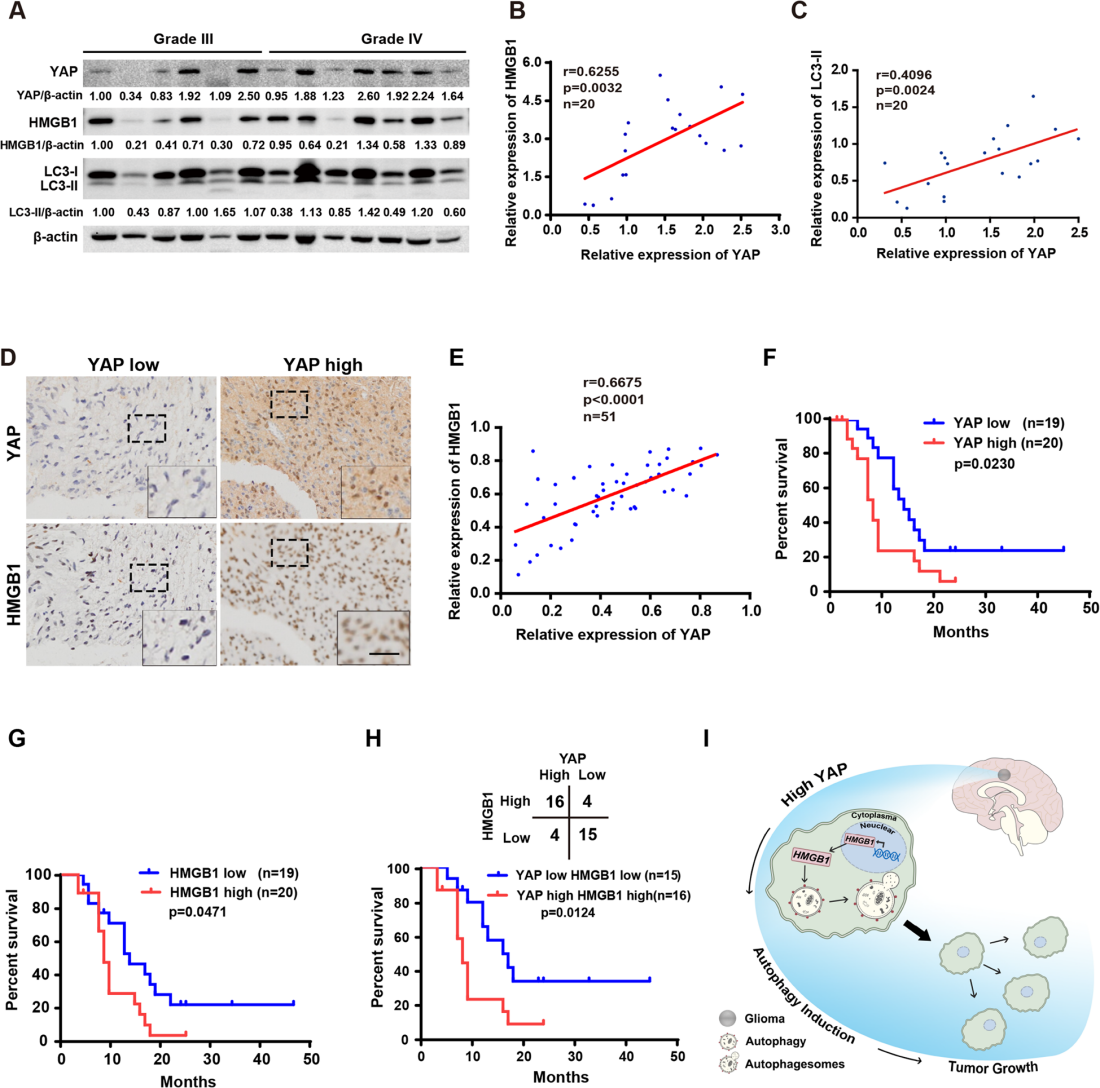
Correlative expression of YAP and HMGB1 are prognosis for clinical GBM. **a** Representative immunoblots of total lysates extracted from high grade samples probed with indicated antibodies. **b** & **c** The protein level of YAP was positively correlated with HMGB1 and LC3-II in gliomas. **d** Representative images of IHC staining of YAP and HMGB1 in clinical GBM samples. Scale bar: 25 μm. **e** The protein level of YAP was positively correlated with HMGB1 in gliomas examined by IHC. **f-h** Kaplan-Meier analyses for GBM patients with high or low level of YAP (**f**) or HMGB1 (**g**) or with YAP and HMGB1 both high or low expression (down of **h**). Correlations of IHC data for high or low YAP expression relative to level of HMGB1 (up of **h**). The cut-off to define high or low expression was the median value. **i** Working model: YAP promotes glioma autophagy and progression by promoting the transcription and translocation of HMGB1 from nucleus to cytoplasm, leading to autophagy

Next, examined by western blotting, we found a positive correlation between YAP and HMGB1, YAP and LC3-II expression in high grade glioma samples, in line with CGGA database analysis (Fig. 7a-c). To address the importance of YAP and HMGB1 to GBM biological behavior further, we analyzed their expression in 51 High Grade Glioma samples by immunohistochemical staining (Fig. 7d) and found a positive correlation between YAP and HMGB1 expression (Fig. 7e, h up), in line with the above results. According to the data of High Grade Glioma patients with follow-up information (*n* = 39), the clinical implications of these findings were highlighted by the determination of the survival time of patients being inversely correlated with YAP and HMGB1 staining (Fig. 7f-h). Most interestingly, patients with YAP and HMGB1 both high showed poorer prognosis (Fig. 7h).

## Discussion

Numerous studies have provided compelling evidence that malignant gliomas are the most common type of primary brain tumor with a median survival not exceeding 15 months [32]. Glioma progression is closely related to cell autophagy under the condition of hypoxia and starvation in brain. Here, we demonstrated that YAP promotes glioma autophagy and then progression both in vitro and in vivo. Mechanistically, YAP performed the above effect by promoting the transcription and translocation of HMGB1 from nucleus to cytoplasm, leading to autophagy (Fig. 7i). Furthermore, the expression of YAP and HMGB1 were positively correlated with each other and suggested poor prognosis for clinical GBM. In conclusion, our data describe a YAP-HMGB1 signaling axis that mediates YAP promoting autophagic activity and tumorigenicity in GBM, and provide a basis for the development of alternative strategies, such as combination therapy with autophagy inhibitors for YAP high GBM patients.

The Hippo/YAP pathway, originally identified as a central developmental regulator of organ size, has been found perturbed in many types of human tumors. Standing at the centrepiece of the signaling hub, YAP captures information from the physical context and turns it into a transcriptional response [33], which therefore takes part in many cell processes, such as tumor growth, survival, metastasis, stemness, drug resistance and autophagy. YAP has been reported to protect MCF7 cells from apoptosis under ND conditions by enhancing autophagic flux [20]. In this study, we found that YAP promotes glioma autophagy not only under basal conditions, but also under stress-induced conditions. Recent phase I/II clinical trials for GBM using autophagy inhibitor CQ or HCQ in combination with other drugs or therapeutic modalities, have shown some encouraging results [8, 34]. We found that inhibiting autophagy by CQ partially abolished the promoting effect of YAP on glioma progression, indicating that the development of YAP high gliomas partially depends on autophagy driven by YAP. However, as for why the survival benefit of CQ treatment was smaller than the effect on growth inhibition, we presently have no good explanation. We guess it may be related to the dose of CQ used and some unknown reasons. Our study advanced the knowledge of the association of YAP with GBM and autophagy.

Since YAP is a famous co-transcription factor, all studies about its molecular mechanism on autophagy focus on its transcription role. For example, Song et al. reported that the regulation of YAP on autophagy depends on the TEAD family of growth-promoting transcription factors [20]. Recent studies showed that YAP regulates autophagy in breast cancer cells via promoting transcription of myosin-II family genes and Armus [35, 36]. In this study, by using iTraq based proteomics analysis and following systemic experiments, we identified that HMGB1 was the mediator of YAP on autophagy. HMGB1 has been previously linked to the induction of autophagy and thought to be a potential for exploiting HMGB1-induced autophagy in cancer therapy [26, 37]. Notably, Zhang et al. found that HMGB1-TLR2 induced CD133^−^ cancer cells dedifferentiation via regulating Hippo/YAP pathway [38]. Chen et al. also found that HMGB1 controls liver cancer initiation through YAP-dependent aerobic glycolysis [39]. The above studies reported that HMGB1 regulates YAP activity. Here, we reported that YAP upregulated HMGB1 by promoting its transcription but not by affecting its posttranslational modification. In addition, YAP promotes the translocation of HMGB1 from the nucleus to cytoplasm, leading to autophagy. These results highlighted YAP-HMGB1 pathway as a crucial mediator of YAP-induced autophagy in GBM cells. Furthermore, although Su et al. reported that HMGB1 increased after nutrient depletion in Lewis cells [40], we have not find the HMGB1 increase in U251 cells exposed to autophagic conditions in our system. Tang et al. [26] and Thorburn et al. [41] have also did not find HMGB1 increase in total cell lysate in MEF and U87 cells after autophagy induction, in line with ours. Both groups just found the translocation of HMGB1 from nucleus to external or to cytoplasma of cells after H2O2 or HBSS induction. We deduce that the difference may be caused by different cell lines used and different way of autophagy induction.

Key functions of autophagy are to provide energy and metabolic precursors under conditions of starvation and to alleviate stress by removal of damaged proteins and organelles, which are deleterious for cell survival. Therefore, autophagy appears to serve as a pro-survival stress response in most settings. Generally speaking, in established tumor, by enhancing stress tolerance and providing more nutrient and energy, autophagy plays important roles in supporting tumor cell survival [42]. Glioma, which grows in a hypoxic and hypoglycemic environment for long time, is a good model for studying autophagy and its role in cancer development. In our system, by using three cohorts of glioma samples, we found that YAP and HMGB1 expression is positively correlated with each other, and were prognostic for clinical GBM, in line with our results in vitro. These data suggest that YAP and HMGB1 are two feasible therapeutic targets for malignant human cancers treatment such as GBM.

## Conclusion

Our data indicates that YAP induces cytoprotective autophagy and contributes to GBM malignancy through upregulating HMGB1. Since preclinical studies involving autophagy inhibition [43–45] have motivated a large number of clinical trials targeting autophagy as part of combination therapy treatment of various cancers, this study revealed a clinical opportunity involving the combination of chemo-radiotherapy with pharmacological autophagy inhibition for treating GBM patients with YAP high expression. It will be of interest to discover whether these results are generalizable to other cancers.

## Supplementary Information

**Additional file 1 **: **Movie 1**. vector group.

**Additional file 2 **: **Movie 2**. YAP over-expression group. After having been transfected with pEGFP-LC3 for 24 h, vector and YAP-overexpression cells were cultured in chambers on the platform of EVOS FL Auto cell imaging system and treated with Rap (200 nM). Images of each group with four randomly selected fields were collected as one frame per 15 min and the recording last for 6 h to make movie.

**Additional file 3 **: **sFig. 1.** YAP upregulates HMGB1 not through posttranslational way. **A&B** Representative immunoblots of total lysates extracted from YAP over-expression or vector cells with or without MG132 (the proteosome inhibitor) treatment in U251 (**A**) and U87 (**B**) glioma cells. **C&D** Representative immunoblots of total lysates extracted from YAP over-expression or vector cells with or without chloroquine (the autophagy inhibitor) treatment in U251 (**C**) and U87 (**D**) glioma cells.

**Additional file 4 **: **sFig. 2.** Expression of YAP and HMGB1 in glioma tissues. Representative immunoblots of total lysates extracted from nontumor or different grade glioma tissues probed with indicated antibodies. β-actin served as the protein loading control.

**Additional file 5 **: **sFig. 3.** Generation of HMGB1 down-regulation glioma cells. Representative images showing the high infection efficiency of three HMGB1 shRNAs in U87 cells. Scale bar: 100 μm.

**Additional file 6 **: **sFig. 4.** Clinical relevance of YAP, HMGB1 and LC3-II in gliomas. **A&B.** Association of YAP expression patterns with overall survival time were presented by Kaplan-Meier plotter based on the TCGA and CGGA database. *p* < 0.0001. **C.** Association of HMGB1 expression patterns with overall survival time in high grade glioma were presented by Kaplan-Meier plotter based on the CGGA database. *p* < 0.05.

## Data Availability

All data used in this study are included within the article and additional files.

## Abbreviations

YAP: Yes-associated protein
HMGB1: High mobility group box 1
GBM: Glioblastoma
TEM: Transmission electron microscopy
CQ: Chloroquine
Rap: Rapamycin
ND: Nutrient deprivation
TCGA: The Cancer Genome Atlas
CGGA: The Chinese Glioma Genome Atlas
LC3B: Microtubule-associated protein 1 light chain
AVs: Autophagic vesicles
APs: Autophagosomes
ALs: Autolysosomes
iTraq: Isobaric tags for relative and absolute quantification
