# Supplementary figures and images for: YAP promotes autophagy and progression of gliomas via upregulating HMGB1
Source: J Exp Clin Cancer Res. 2021 Mar 16;40:99. doi: 10.1186/s13046-021-01897-8 (PMC7968184; doi:10.1186/s13046-021-01897-8)

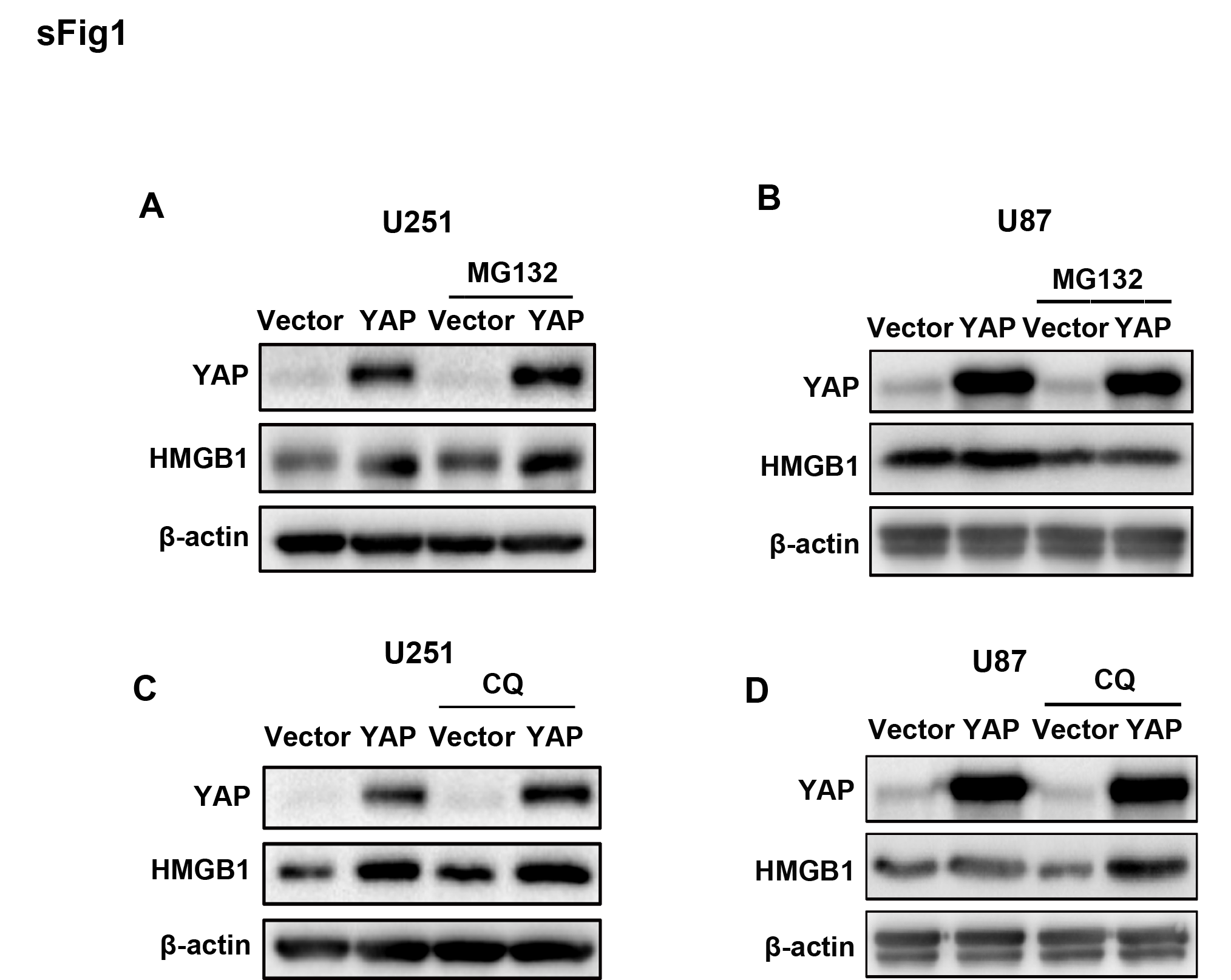

Supplement: Supplementary file 3 — Additional file 3 : sFig. 1. YAP upregulates HMGB1 not through posttranslational way. A&B Representative immunoblots of total lysates extracted from YAP over-expression or vector cells with or without MG132 (the proteosome inhibitor) treatment in U251 (A) and U87 (B) glioma cells. C&D Representative immunoblots of total lysates extracted from YAP over-expression or vector cells with or without chloroquine (the autophagy inhibitor) treatment in U251 (C) and U87 (D) glioma cells. [file 13046_2021_1897_MOESM3_ESM.tif]

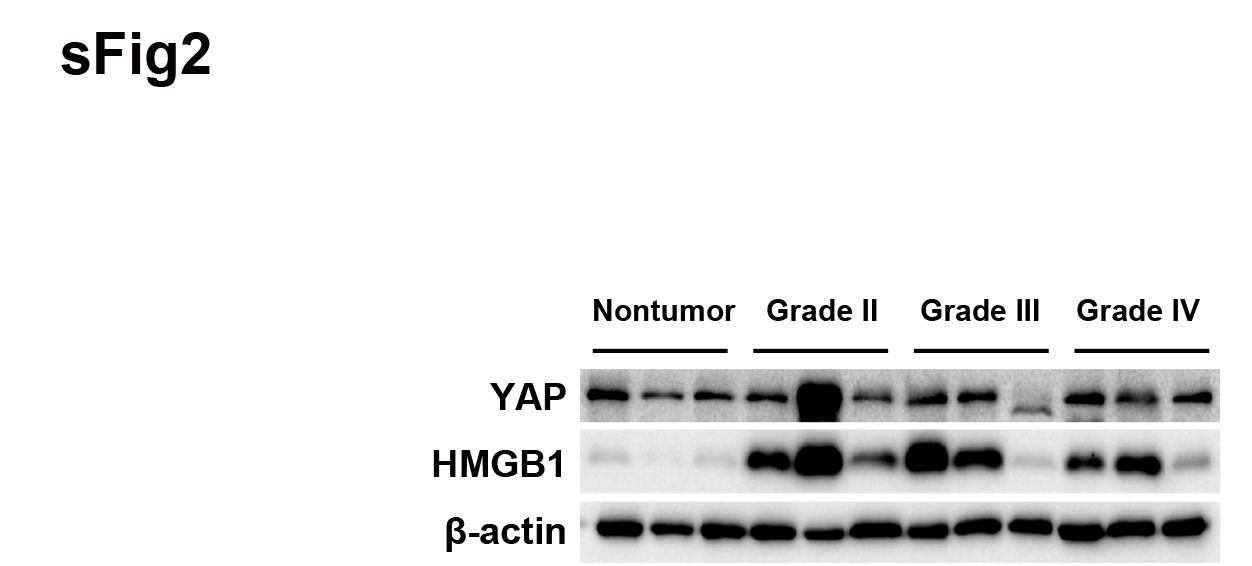

Supplement: Supplementary file 4 — Additional file 4 : sFig. 2. Expression of YAP and HMGB1 in glioma tissues. Representative immunoblots of total lysates extracted from nontumor or different grade glioma tissues probed with indicated antibodies. β-actin served as the protein loading control. [file 13046_2021_1897_MOESM4_ESM.tif]

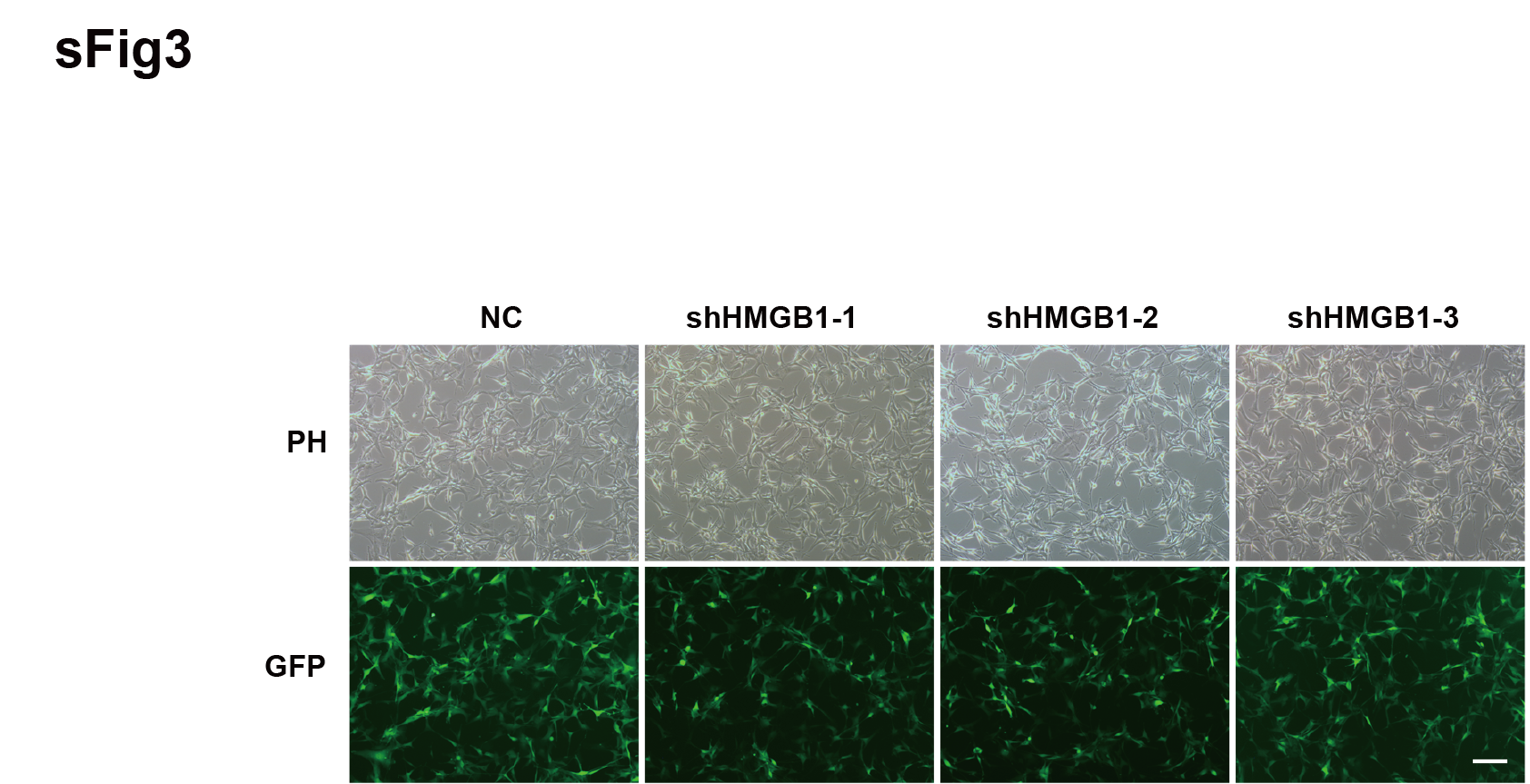

Supplement: Supplementary file 5 — Additional file 5 : sFig. 3. Generation of HMGB1 down-regulation glioma cells. Representative images showing the high infection efficiency of three HMGB1 shRNAs in U87 cells. Scale bar: 100 μm. [file 13046_2021_1897_MOESM5_ESM.tif]

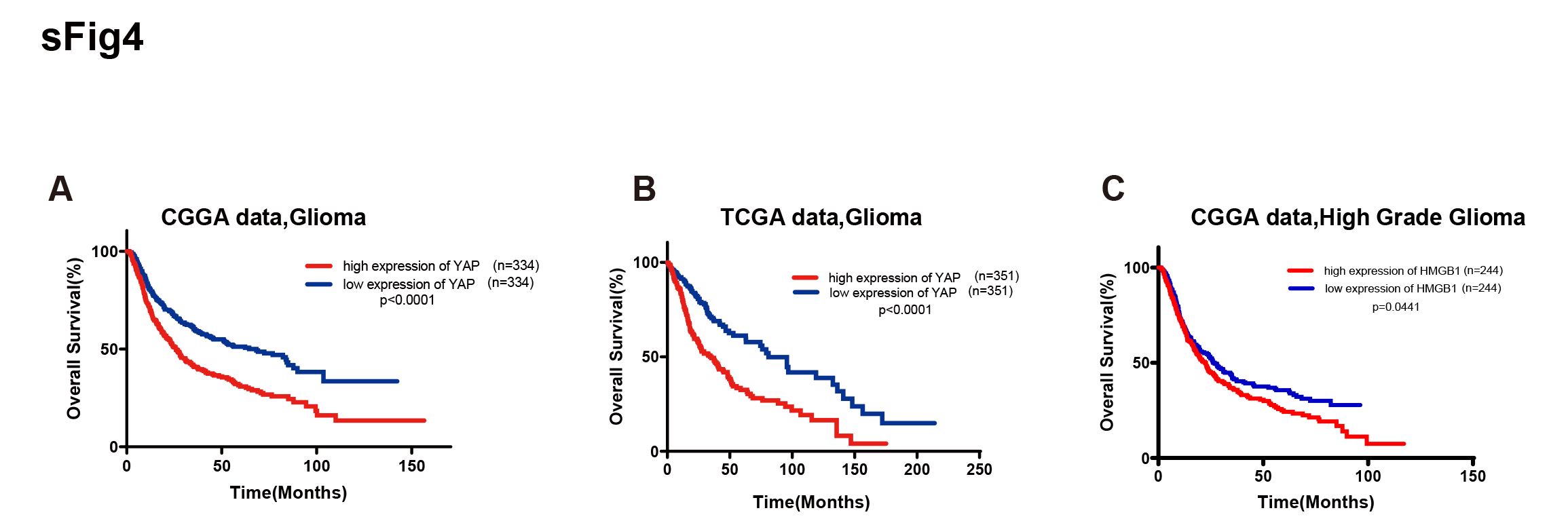

Supplement: Supplementary file 6 — Additional file 6 : sFig. 4. Clinical relevance of YAP, HMGB1 and LC3-II in gliomas. A&B. Association of YAP expression patterns with overall survival time were presented by Kaplan-Meier plotter based on the TCGA and CGGA database. p < 0.0001. C. Association of HMGB1 expression patterns with overall survival time in high grade glioma were presented by Kaplan-Meier plotter based on the CGGA database. p < 0.05. [file 13046_2021_1897_MOESM6_ESM.tif]
